# Pacemaker Lead Perforation Leading to Cardiac Tamponade and Subsequent Anterior STEMI

**DOI:** 10.1016/j.jaccas.2026.108324

**Published:** 2026-05-14

**Authors:** John Martinez-Ponce, Oskar Ubysz, Anthony Costa, Lina Ataya, Sandeep Bhangu, Thomas Vanhecke

**Affiliations:** a Department of Internal Medicine, Henry Ford Genesys, Grand Blanc, Michigan, USA; b Department of Cardiology, Henry Ford Genesys, Grand Blanc, Michigan, USA

**Keywords:** cardiac pacemaker, myocardial infarction, pericardial effusion, tamponade

## Abstract

**Background:**

Pacemaker lead perforation is an uncommon but potentially fatal complication of cardiac implantable electronic devices, particularly when hemopericardium and tamponade occur in patients requiring antithrombotic therapy.

**Case Report:**

A 74-year-old woman with atrial fibrillation on apixaban and a recent dual-chamber pacemaker implantation presented with dizziness, falling, and dysarthria; her brain computed tomography was negative. She developed syncope with a 6-second ventricular pause and enlarging pericardial effusion and an emergent surgical pericardial window with drainage of 350 mL of hemorrhagic fluid, confirming right ventricular lead perforation left in situ. Shortly after stabilization, she developed anterior ST-segment elevation myocardial infarction because of proximal left anterior descending artery occlusion that was treated with percutaneous coronary intervention. Echocardiogram showed a left ventricular ejection fraction of 35% to 40% without recurrent effusion.

**Conclusions:**

Early recognition, prompt drainage, and careful balancing of bleeding and thrombosis risks are essential; multidisciplinary management enables favorable outcomes.

**Take-Home Message:**

Maintain suspicion for lead perforation despite a normal device interrogation, and act rapidly in the event of tamponade while coordinating multidisciplinary care.

## Graphical Abstract

**Figure:**
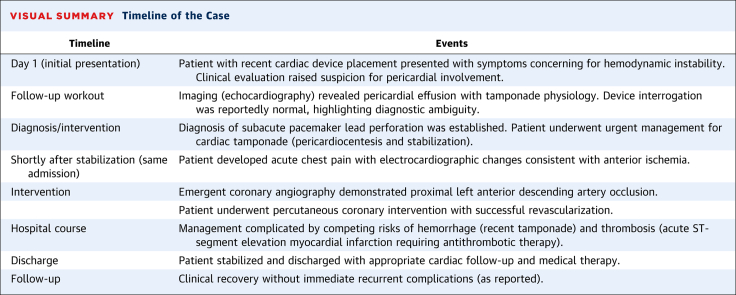

Pacemaker or implantable cardioverter-defibrillator (ICD) lead perforation is an uncommon but life-threatening complication of cardiac implantable electronic devices (CIEDs) that demands rapid recognition and decisive management to prevent hemodynamic collapse from hemopericardium and cardiac tamponade.^1^ Although the overall incidence is low, approximately 0.1% to 0.8% for pacemaker leads and up to 5.2% for ICD leads, the increasing number of CIED implantations worldwide means even rare events will be encountered more frequently in clinical practice.^2^ Leadless pacemaker systems are not exempt, with early data suggesting perforation rates of approximately 1% to 1.5%, potentially reflecting larger delivery systems and operator learning curves.^3^

**Take-Home Message**

• This case highlights the importance of maintaining a high suspicion for lead perforation in postimplant patients with unexplained symptoms, recognizing that normal device interrogation does not exclude perforation, and acting rapidly in cases of cardiac tamponade.
• It also underscores the need to carefully balance bleeding and thrombosis risks while using a multidisciplinary approach involving cardiology, electrophysiology, and cardiothoracic surgery.

Several factors increase vulnerability to perforation. Most events occur at the apex of the right ventricle, a commonly used implantation site with thinner myocardium and greater susceptibility to full-thickness penetration.^4^ In contrast, septal or right ventricular outflow tract positioning appears to be implicated less frequently and may offer relative protection.^5^ Device-related factors are also relevant: Active-fixation leads have been associated with a higher perforation risk than passive leads, particularly when excessive torque is applied.^6^ Additionally, smaller-diameter or stiffer leads may increase focal pressure at the lead–myocardial interface.^7^ Patient-related risk factors include advanced age, female sex, low body mass index, corticosteroid exposure, and periprocedural anticoagulation, which may increase bleeding risk if perforation occurs.^7^^,^^8^

Clinical presentation is heterogeneous and may occur acutely (within 24 hours), subacutely (days to weeks), or in a delayed fashion months to years after implantation.^9^ Symptoms may include chest pain, dyspnea, syncope, diaphragmatic irritation, or device malfunction, although some cases remain asymptomatic. Notably, imaging studies suggest that clinically silent microperforations may be under-recognized. Transthoracic echocardiography is the first-line diagnostic modality, with cardiac computed tomography providing high sensitivity when the diagnosis is uncertain. We report a case of subacute right ventricular lead perforation complicated by cardiac tamponade, followed by acute anterior ST-segment elevation myocardial infarction (STEMI), highlighting diagnostic challenges and management complexity when hemorrhagic and ischemic risks intersect.

## Case Presentation

A 74-year-old woman with hypertension, paroxysmal atrial fibrillation on apixaban, sick sinus syndrome with intermittent complete heart block (status post dual-chamber pacemaker implantation < 1 week prior), prior transient ischemic attack, and remote subarachnoid hemorrhage presented after a fall associated with dizziness, slurred speech, and difficulty completing sentences. Initial computed tomography of the brain showed no acute pathology. Thrombolysis was contraindicated, and magnetic resonance imaging was deferred because of recent pacemaker implantation. Neurology considered transient ischemic attack vs concussion or seizure without definitive diagnosis.

While hospitalized, the patient developed abrupt syncope. Telemetry revealed bradycardia with intermittent high-grade atrioventricular block and a 6-second ventricular pause. Device interrogation demonstrated normal dual chamber pacing, dual chamber sensing, dual response, rate modulation function. A 12-lead electrocardiogram showed normal sinus rhythm with a narrow QRS complex and no ischemic changes (Figure 1). Transthoracic echocardiography revealed preserved left ventricular function (ejection fraction: 60%-65%) and a small pericardial effusion. Troponin levels were mildly elevated.

**Figure 1:**
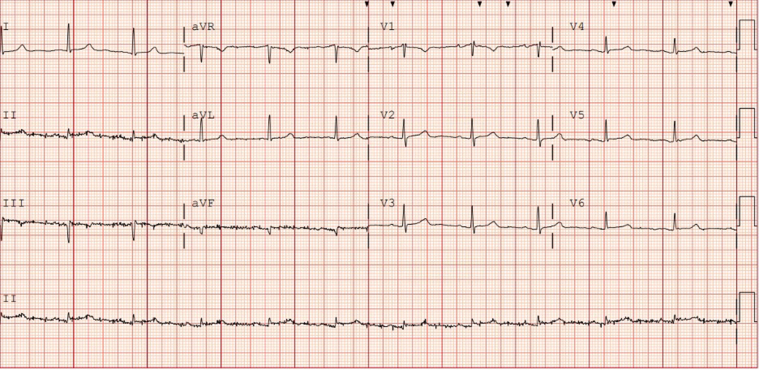
Normal Sinus Rhythm, Left-Axis Deviation, With No ST-Segment Changes Noticed A baseline electrocardiogram demonstrating normal sinus rhythm without ST-segment abnormalities after a syncopal episode.

Over the next several hours, the patient developed hypotension, chest discomfort, acute kidney injury, anemia, and elevated lactate levels. Repeat imaging demonstrated an enlarging pericardial effusion. Echocardiography confirmed tamponade physiology with right ventricular diastolic collapse and respiratory variation in transvalvular flow (Figure 2). Given the patient’s hemodynamic instability, an urgent surgical pericardial window was performed, draining approximately 350 mL of hemorrhagic fluid. Intraoperative findings revealed a right ventricular lead perforation near the apex. The lead was left in situ.

**Figure 2:**
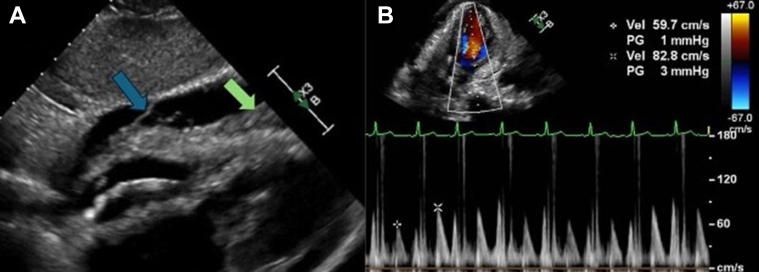
Transthoracic Echocardiogram Demonstrating Moderate Pericardial Effusion With Right Ventricular Diastolic Collapse and Features Consistent With Early Cardiac Tamponade (A) Subxiphoid echocardiogram window showing a moderately sized inferior pericardial effusion with fibrous stranding (blue arrow) and a forming thrombus (green arrow). (B) Apical echocardiogram window showing mitral inflow pulse-wave Doppler with 28% E-wave variation between inspiration and expiration.

After stabilization, the patient developed new chest pain. Electrocardiography demonstrated ST-segment elevations in leads V_1_ and V_2_ (Figure 3) with concordant T-wave changes (Figure 4). A bedside echocardiography revealed new hypokinesis of the anterior and apical segments. Emergent coronary angiography demonstrated a 100% thrombotic occlusion of the proximal left anterior descending artery (Figure 5). Primary percutaneous coronary intervention with drug-eluting stents restored coronary flow. Intraprocedural transesophageal echocardiography confirmed a stable lead position without recurrent effusion.

**Figure 3:**
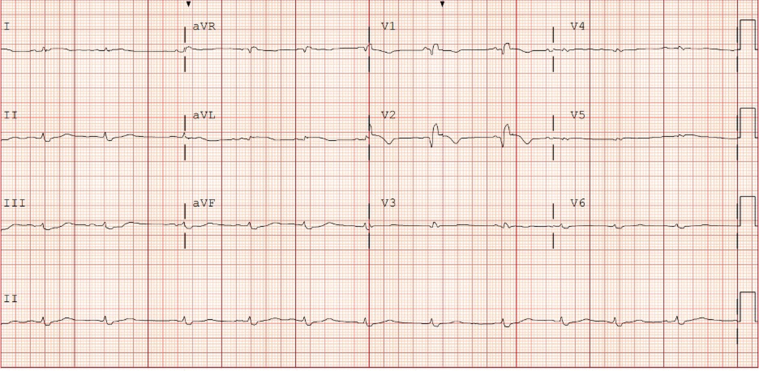
ST-Segment Elevation With Concordant T-Wave Inversion Electrocardiogram demonstrating anterior ST-segment elevations in leads V_1_ and V_2_ consistent with acute anterior ST-segment elevation myocardial infarction.

**Figure 4:**
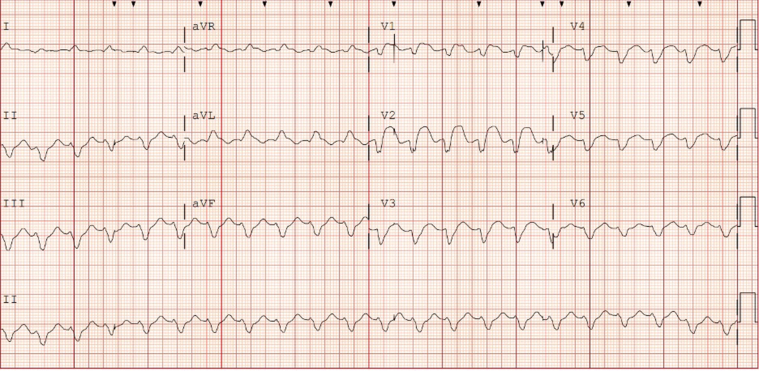
Dynamic Evolution of Anterior Ischemic Changes Serial electrocardiogram demonstrating dynamic evolution of anterior ischemic changes and worsening ST-segment elevation on V_1_ to V^3^, with no demonstration of a wide QRS complex.

**Figure 5:**
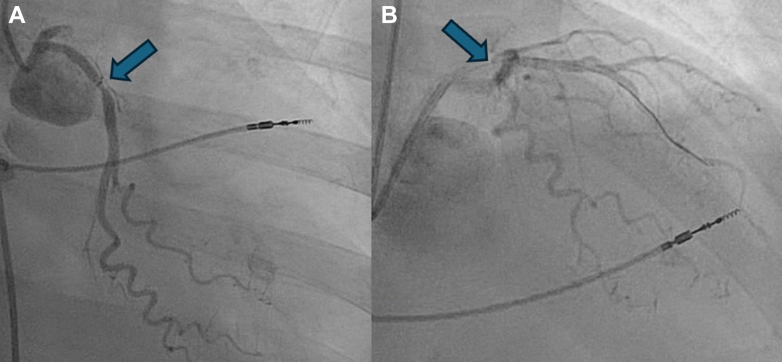
Coronary Angiography Demonstrating Complete Thrombotic Occlusion of the Proximal Left Anterior Descending Artery Before Intervention (A) Left heart catheterization in the right anterior oblique caudal view showing proximal left anterior descending artery occlusion. (B) Post-thrombus wiring right anterior oblique caudal view showing mid– left anterior descending artery thrombus in addition to the proximal lesion.

Postprocedurally, the patient developed ventricular arrhythmias, including nonsustained ventricular tachycardia and sustained monomorphic ventricular tachycardia, consistent with reperfusion arrhythmia, managed with amiodarone. Repeat echocardiography demonstrated reduced left ventricular ejection fraction (35%-40%) with anterior wall akinesis. The pericardial effusion did not recur. The patient was discharged on guideline-directed medical therapy.

## Discussion

This case illustrates a rare but severe complication of CIED implantation, lead perforation with cardiac tamponade, followed by acute myocardial infarction, presenting a complex interplay between bleeding and thrombosis.^1^^,^^2^ Although the association between these events is notable, a direct causal relationship cannot be definitively established, and their coexistence may reflect coincidence rather than causation.

### Lead perforation: risk and recognition

Lead perforation is infrequent but potentially catastrophic, with reported incidences ranging from 0.1% to 0.8% for pacemakers and up to 5.2% for ICDs.^1^ This case emphasizes that clinically significant perforation may present outside the immediate postimplant period and with nonspecific findings. Although most cases occur within 24 hours of implantation, subacute and delayed presentations are increasingly recognized.^8^^,^^9^ The right ventricular apex is particularly vulnerable because of its relatively thin myocardium, especially in elderly and low-body-weight patients.^3^^,^^4^ The key challenge illustrated here is that device interrogation may remain normal despite perforation, delaying recognition. The patient's presentation underscores the importance of maintaining suspicion in the setting of unexplained hypotension, chest pain, or evolving pericardial effusion after implantation, even when initial device parameters are reassuring.

### Diagnostic approach

Transthoracic echocardiography is the imaging modality of choice for assessing pericardial effusion and tamponade physiology.^2^ However, cardiac computed tomography provides superior visualization of lead position and is particularly useful in equivocal cases or in hemodynamically stable patients.^8^

### Management considerations

The primary priority in this case was hemodynamic stabilization in the setting of tamponade. Surgical intervention was favored over percutaneous drainage because of the presence of a loculated hemorrhagic effusion and recent device implantation, illustrating the need to individualize management based on anatomical and procedural factors.^2^ Lead management similarly required a nuanced approach; in select cases such as this, leaving the lead in situ may mitigate the risk of worsening perforation or bleeding.^9^ This reinforces that management decisions must balance procedural risks with hemodynamic stability rather than follow a uniform algorithm.

### Intersection with acute coronary syndrome

The subsequent STEMI presented a unique clinical challenge because the need for dual-antiplatelet therapy and anticoagulation increases bleeding risk in a patient with recent hemopericardium. Potential mechanisms for STEMI in this setting include a prothrombotic state linked to acute illness, which can be triggered or revealed by physiological stress and systemic inflammatory and prothrombotic activation; hemodynamic stress and demand ischemia from tamponade physiology (hypotension with reduced coronary perfusion pressure and catecholamine surge), potentially converting stable left anterior descending artery disease into transmural ischemia^1^^,^^2^; or, more rarely, direct CIED-related coronary impairment (eg, right ventricular lead impingement or perforation with left anterior descending artery spasm, dissection, or extrinsic compression), which has been reported to cause anterior STEMI and malignant ventricular arrhythmias.^10^ However, a direct mechanistic link between lead perforation and myocardial infarction remains speculative, and it is equally plausible that the STEMI happened independently of the perforation. Regardless of the mechanism, management depends on sequencing: Life-threatening tamponade requires urgent drainage and hemostasis before escalation of antiplatelet or anticoagulant therapy, and, once stabilized, guideline-directed emergent coronary angiography and percutaneous coronary intervention should proceed with bleeding-avoidance strategies and close monitoring for recurrent pericardial bleeding.

### Arrhythmias and post–myocardial infarction course

Reperfusion arrhythmias are common after STEMI and were observed in this patient. These are typically transient and respond to antiarrhythmic therapy and electrolyte optimization.

## Conclusions

Pacemaker lead perforation is a rare but life-threatening complication requiring rapid recognition and intervention. This case highlights the importance of early diagnosis, prompt hemodynamic stabilization, and individualized management strategies. The coexistence of cardiac tamponade and acute myocardial infarction underscores the complexity of balancing hemorrhagic and ischemic risks and emphasizes the need for multidisciplinary care.

## Funding Support and Author Disclosures

The authors have reported that they have no relationships relevant to the contents of this paper to disclose.

**Visual Summary:** Timeline of the Case

Timeline	Events
Day 1 (initial presentation)	Patient with recent cardiac device placement presented with symptoms concerning for hemodynamic instability. Clinical evaluation raised suspicion for pericardial involvement.
Follow-up workout	Imaging (echocardiography) revealed pericardial effusion with tamponade physiology. Device interrogation was reportedly normal, highlighting diagnostic ambiguity.
Diagnosis/intervention	Diagnosis of subacute pacemaker lead perforation was established. Patient underwent urgent management for cardiac tamponade (pericardiocentesis and stabilization).
Shortly after stabilization (same admission)	Patient developed acute chest pain with electrocardiographic changes consistent with anterior ischemia.
Intervention	Emergent coronary angiography demonstrated proximal left anterior descending artery occlusion.
	Patient underwent percutaneous coronary intervention with successful revascularization.
Hospital course	Management complicated by competing risks of hemorrhage (recent tamponade) and thrombosis (acute ST-segment elevation myocardial infarction requiring antithrombotic therapy).
Discharge	Patient stabilized and discharged with appropriate cardiac follow-up and medical therapy.
Follow-up	Clinical recovery without immediate recurrent complications (as reported).

## References

[1] Refaat M.M., Hashash J.G. (2010). Late perforation by cardiac implantable electronic device leads. Int J Cardiol.

[2] Kusumoto F.M., Schoenfeld M.H., Wilkoff B.L. (2017). 2017 HRS expert consensus statement on CIED lead management and extraction. Heart Rhythm.

[3] Vanezis A.P., Prasad R., Andrews R. (2017). Pacemaker leads and cardiac perforation. JRSM Open.

[4] Cano Ó., Osca J., Sancho-Tello M.J. (2017). Incidence and predictors of clinically relevant cardiac perforation after active-fixation lead implantation. Europace.

[5] Chao J.A.K., Firstenberg M.S. (2017). Delayed pacemaker lead perforations: diagnosis and management. J Geriatr Cardiol.

[6] Vamos M., Erath J.W., Benz A.P. (2017). Incidence of cardiac perforation with conventional and with leadless pacemaker systems: a systematic review and meta-analysis. J Cardiovasc Electrophysiol.

[7] Ngo L., Nour D., Denman R.A. (2021). Safety and efficacy of leadless pacemakers: a systematic review and meta-analysis. J Am Heart Assoc.

[8] Hirschl D.A., Jain V.R., Spindola-Franco H., Gross J.N., Haramati L.B. (2007). Prevalence and characterization of asymptomatic pacemaker and ICD lead perforation on CT. Pacing Clin Electrophysiol.

[9] Simsolo E., Wilkoff B.L. (2022). A shocking case of pacemaker lead perforation. JACC Case Rep.

[10] Byrne R.A., Rossello X., Coughlan J.J. (2023). 2023 ESC Guidelines for the management of acute coronary syndromes. Eur Heart J.

